# Coat Color and Cat Outcomes in an Urban U.S. Shelter

**DOI:** 10.3390/ani10101720

**Published:** 2020-09-23

**Authors:** Robert M. Carini, Jennifer Sinski, Jonetta D. Weber

**Affiliations:** 1 Department of Sociology, University of Louisville, Louisville, KY 40292, USA; jonettaweber@louisville.edu; 2 Department of Sociology, Bellarmine University, Louisville, KY 40205, USA; jsinski2@bellarmine.edu

**Keywords:** shelter euthanasia, shelter adoption, rehoming, coat color, black cat, Halloween

## Abstract

**Simple Summary:**

There is continuing debate as to whether individuals prefer companion cats of varying coat colors, and if so, how color preferences may affect whether cats in shelters are euthanized, adopted, or transferred to another organization. This study analyzed outcomes for nearly 8000 cats admitted to an urban public shelter in Kentucky, USA from 2010 through 2011. While coat color overall was not an important predictor of cat outcomes, a tiered pattern among particular colors was detected. Specifically, black and white cats experienced the highest and lowest chances of euthanasia, respectively, while brown and gray cats experienced more middling chances. Orange cats’ relative chances of euthanasia were more difficult to gauge, but orange and white cats had similar euthanasia and adoption outcomes in the most nuanced model. In addition, there has been persistent speculation that the public’s interest in—and preference for—black cats might spike before Halloween due to cats’ associations with the holiday. However, the present study found that a subsample of more than 1200 entirely black cats did not experience improved chances of adoption or transfer to a rescue organization in October compared to other months. Overall, this study provides weak evidence for what has been termed “Black Cat Bias” by others, and hints that black cats in public shelters should receive extra consideration for rehoming.

**Abstract:**

Some nonhuman animal shelters have developed rehoming programs for black cats to remedy what they believe are their higher rates of euthanasia and lower rates of adoption. This study reviews humans’ preferences/aversions to cats of various coat colors and uses contingency tables and multinomial logistic regression to test possible differences in outcomes (euthanasia, adoption, or transfer) for 7983 cats that entered an urban public shelter in Kentucky, USA from 2010 through 2011. While coat color overall was negligibly associated with cat outcomes in a contingency table, the pairwise difference between black and white cats was significant (*p* < 0.05) and nontrivial in strength. Specifically, black cats experienced the highest euthanasia and lowest adoption rates, while white cats had the lowest euthanasia and highest adoption rates. Brown, gray, and orange cats experienced similar outcomes, but middling between those of black and white cats. These patterns by color remained weak but significant after controlling for breed and stray status in regression analysis, with the exception of orange and white, which did not differ significantly. A subsample of 1219 entirely black cats was analyzed to assess whether they had different outcomes during the run-up to Halloween; their October percentages of adoption and transfer were comparable to or lower than all other months of the calendar year. Thus, this study did not find that outcomes improved for black cats during October. Overall, this study provides weak support for what has been termed “Black Cat Bias” by others, and hints that black cats in public shelters should receive extra consideration for rehoming, particularly if such efforts do not substantially redirect resources from other initiatives.

## 1. Introduction

The American Society for the Prevention of Cruelty to Animals (ASPCA) estimates that 3.2 million cats entered shelters and 860,000 were euthanized from 2015 to 2018 in the USA [1]. Despite decreased euthanasia rates for cats in the USA in recent years [1,2], unwanted companion cats continue to present a vexing problem for shelters, adoption agencies, and society at large. As the United States continues to experience a paradigm shift from animal as objects to animal as subjects, euthanasia of healthy companion animals for reasons of time and space is becoming less acceptable as a response to the social problem of pet overpopulation [3]. Complicating efforts to reduce both shelter intake and euthanization numbers of companion cats is the fact that these statistics are difficult to assess due to a lack of consistent record keeping and reporting [4]. A primary difficulty to conducting research on factors that sway adoption decisions is that data often hinge on the cooperation of the animal sheltering industry itself. Societal disagreement over how to address the problem of pet overpopulation, including the euthanasia of healthy animals, prompts many shelters to resist sharing their data with academic researchers [5,6,7].

Efforts to decrease the number of euthanized cats have been multipronged: shelters and adoption agencies often promote spaying/neutering, make greater attempts to reunite lost cats with their owners, and implement rehoming programs. For their part, researchers have conducted studies to better understand the adoption preferences of potential owners, including identifying cats’ physical characteristics and traits, as well as shelter factors, that shape cats’ outcomes for the better or worse, such as sex and age [8,9], breed [9], stray vs. owner surrender [10], activity level [11], housing conditions and handling procedures [12], and shelter or cage enrichment experiences [9,13], among others.

One characteristic of particular interest is that of coat color or shade. Black animals, particularly cats and dogs, have often been maligned in Western literature and popular culture [14]. For instance, black cats were often associated with witches in medieval Europe; Linton [15] (p. 64) noted that “…[black] cats were the familiars of witches, or even the witches themselves, since it was commonly believed that witches often transformed themselves into cats.” Given their association with witches and the devil, black cats—and even their owners—sometimes endured acts of cruelty or were killed [16]. In some parts of the world, black cats are thought to bring good fortune [17], while in others, including the USA, they are portrayed as symbols of the supernatural and evil, and thought to auger bad luck. Those who express biased attitudes toward photographs of black cats have been found to be more likely to hold a variety of superstitious beliefs [18]. Further, the notion that black cats portend ill fortune forms the basis for an item in the Paranormal Belief Scale, an instrument that attempts to operationalize belief in superstition as a subscale [19]. Such metaphors and beliefs may reinforce negative perceptions of black cats and provide a basis for stereotyping [20], as well as perpetuate the notion that they are at risk of being adopted for nefarious reasons during Halloween given that they have become an enduring symbol of the holiday—despite a paucity of empirical evidence to suggest that black cats endure any worse treatment at Halloween [21].

Apart from any holiday connection, black cats are considered by some shelters and shelter administrators to be less likely to be adopted at any time throughout the year, thus suffering from “Black Cat Bias” (BCB) [18]. Black pets may not fare as well because of negative media portrayals and a greater difficulty in distinguishing or photographing their facial characteristics [18], particularly in dimly lit facilities [22]. For instance, the Royal Society for the Prevention of Cruelty to Animals has reported that many black cats end up at their facilities after being abandoned by owners because the cats did not photograph well in selfies [23]. Some have effectively widened the BCB concept to include coat shades, terming it “Dark Cat Syndrome” (DCS) [24]. DCS involves cultural and visual preferences favoring cats of lighter coat shades over darker ones, resulting in the disproportionate adoption of lighter-coated cats and euthanization of darker-coated counterparts in shelters [24].

Shelters and rescue organizations have implemented promotions and specific practices to rehome black cats, which implies that many in their leadership/staff believe BCB to be a real phenomenon [25]. For instance, Black Cat Appreciation Day is celebrated annually by the ASPCA to raise the profile of adoptable black cats; the organization has offered a reduced adoption fee for black kittens or a free carrier for adult black cats [26]. Further, animal shelters/organizations (e.g., the Humane Society of the United States and the ASPCA) have developed “best practices” for shelters to increase adoption rates of black animals. Tips include using brightly-colored collars, bows, bandannas, or backgrounds for added contrast against dark fur; providing brightly-colored toys in their enclosures; strategically considering lighting for maximum effect; and using professional photographers [20,27,28,29]; at a minimum, these prescribed practices appear designed to address the visual perception of black cats. While the Humane Society of the United States acknowledges that shelters once pulled black cats from adoption during past Halloween seasons due to fears of nefarious activities toward cats, they now suggest offering adoption events specifically targeting entirely black cats, including “Black (Cat) Friday Deals,” and “Everything Goes with Black” [29].

There is a small body of academic research from several countries that has reported disadvantaged shelter outcomes for black or darker-colored cats, yet some evidence cited for BCB or DCS is ambiguous or poorly documented. For instance, within a large municipal shelter in California, black and brown cats were reported as the least likely to be adopted, while white, gray, and point colorations were the most likely [30]; however, it is unclear whether these darker and lighter colors differed significantly since tabby was used as the reference color and each odds ratio was associated with a 95% confidence interval that included unity, save for black. In another study, it was found that black cats were the most likely to be euthanized at a municipal shelter in Kitchener-Waterloo, Ontario, Canada [31]. In two shelters in Colorado, USA, black cats were adopted most slowly, followed by cats with primarily black coats, and finally, those of other shades [32]. Black and smoke-colored cats were adopted more slowly than cream-colored cats from a shelter in New York State and had fewer daily clicks on Petfinder [33]. In a study involving three cat shelters in the Czech Republic, darker cats experienced longer lengths of stay than either light or medium shade cats [34]. Another study of records obtained from a county, open-admissions, no-kill shelter in New York State found no color differences in length of stay for kittens, yet reported that lighter-colored cats older than three months were adopted more quickly than darker-colored counterparts [8]. Primarily seal-colored cats had the shortest length of stay. Yellow cats had the longest length of stay, but their stays were not dissimilar to those of black, brown, gray, and white cats. However, in a municipal shelter in Guelph, Ontario, Canada, it was found that that black cats were not significantly disadvantaged relative to counterparts of other coat colors in terms of length of stay [9]. One study reported that black cats had shorter stays than their white counterparts in an urban shelter in Sydney, Australia [35]. The authors suggested that advice commonly dispensed in Australia concerning the need to protect white cats from the sun to prevent skin cancer may result in greater reluctance to adopt white cats, while black cats may require fewer precautions on the part of owners. The aforementioned studies relied on data provided by only one to three shelter(s). A final study is noteworthy since its data source covers many more shelters/organizations than those discussed previously; specifically, it analyzed outcomes for nearly 34,000 cats admitted to 39 Royal Society for the Prevention of Cruelty to Animals shelters in Queensland, Australia, but did not find significant differences in outcomes for black, tabby, tortoiseshell, or “other” colors [13].

Given the potentially important consequences that color may have for adoptable companion animals, it is vital to further examine the possibility of BCB as a hindrance to adoption and contributor to higher rates of euthanasia. The present study also tests whether entirely black cats experience better outcomes before Halloween than they do during other months, i.e., their coat color might be more desirable at this time of year due to black cats’ cultural connections with the holiday. The authors are not aware of any study that examines whether shelter outcomes for entirely black cats may follow a distinct pattern during October vis-à-vis the other 11 months of the year.

The aim of this study, then, is to test whether cats’ coat color is related to their shelter outcomes. Specifically, the hypotheses to be tested are:

**Hypothesis** **1** **(H1).** *Cats of different coat colors will exhibit distinct outcomes in shelters.*

**Hypothesis** **2** **(H2).** *Entirely or primarily black cats will be less likely adopted—and more likely euthanized—than cats of other colors.*

**Hypothesis** **3** **(H3).** *Entirely black cats will be more likely to be adopted or transferred—and less likely euthanized—during the month of Halloween than other months.*

## 2. Materials and Methods

The second author submitted an open records request for 2010–2011 to each of the 120 public county shelters listed on the Kentucky Animal Control Advisory Board’s website. Pursuant to Statute 258.119 [36], shelters were required to keep records on each cat for date impounded; location found; sex and spay/neuter status, if known; breed or description and color; and date reclaimed, adopted, or euthanized. While 20 counties responded, a majority of those provided incomplete data: many lacked animal outcomes, one did not include coat color, and several only delivered monthly summaries (for details, see [4,37]). Data were analyzed from the largest urban shelter that responded with data on cats, located in Kentucky, USA. To the authors’ knowledge, the shelter neither offered targeted rehoming programs for cats of any color during the period studied nor restricted the adoption of black cats during October; multiple calls were made to the shelter to confirm this, but no response was obtained.

### 2.1. Analytic Sample

The shelter provided intake information on 9693 cats from January 2010 through December 2011. Cats that were returned to their owner (*n* = 140), released (*n* = 198), or died from causes other than euthanasia (*n* = 436) were excluded from the analysis. Each variable modeled had less than one percent missing values. In addition, 912 cats were recorded by the shelter as calico or tricolor (*n* = 415), tortoiseshell (*n* = 375), or point coloration (*n* = 122); these cats were ultimately dropped from the analysis on grounds that (1) a primary coat color could not be identified with confidence, and (2) incorporating these into an “other” category would result in color categories that lacked mutual exclusivity. Missing values were treated with listwise deletion, leaving 7983 cats with complete and usable information. Age was considered but ultimately not modeled in the study, as ages were known and logged for only 32.7% of felines. A review of age data revealed newborn kittens through cats 18 years of age.

### 2.2. Measures

Cat outcome was assessed with three mutually exclusive paths: adoption, euthanasia, and transfer; these were coded as “1”, “2”, and “3”, respectively. Transfer denotes cats transferred to another organization or fostered. The key predictor, coat color, was based on the cat being entirely or primarily: black, brown, gray, orange, white, or “other.” “Other” was comprised of colors such as blue, cream, green, gold, silver, tan, and yellow, among others. Each color variable was coded as “1” for the presence of the color and “0” for its absence. Measures were also included for two controls: breed and whether labeled a stray at intake. Prior studies found that breed was a predictor of cat outcomes, e.g., long and medium-haired domestics had longer lengths of stay at shelters than short-haired counterparts [9], and “exotic” breeds experienced briefer stays than shorthairs [8,9]. The shelter in this study used labels of domestic shorthair, domestic mediumhair, and domestic longhair to categorize 7660 (96.0%) as nonidentifiable breeds; this study slotted the remainder of the sample into two non-overlapping categories: “Fancy—mix” (*n* = 192) and “Fancy—single breeds” (*n* = 131). See Appendix A for specific breeds coded as “Fancy—single breeds”; Siamese/Balinese and Russian Blue were the most frequent at *n* = 36 and *n* = 21, respectively. Each of the five breed categories was coded as “1” for its presence and “0” for its absence. Cats labeled as strays at intake tend to experience longer lengths of stay [35]; accordingly, those labeled as strays were coded as “1” in this study and "0" otherwise. Approximately 74% of the sample (*n* = 5875) were identified as strays.

### 2.3. Analytic Approach

This study examined the relationship between various coat colors (black, brown, gray, orange, white, and “other”) and three outcomes (euthanasia, adoption, and transfer) for cats during their first visit to a large public shelter. First, a contingency table was used to test for differences in outcomes across coat colors, as well as whether coat colors differed by each of two covariates. Although some prior research focused on coding color as black versus all others [9] or on a darker/lighter continuum [32], the approach here features six discrete color categories. Nonparametric chi-square testing is appropriate given that the three outcomes, coat color, and two controls are categorical [38]. Cramer’s V statistics were reported as effect sizes when statistically significant associations were found between categorical variables in a contingency table with more than two rows and/or columns [38]. Second, multinomial logistic regression was used to test whether coat color predicted cat outcome net of the two controls, breed and stray status. Multinomial logistic regression is an extension of binary regression and is appropriate when modeling three or more unordered and exhaustive categorical outcomes [39]. Each contrast in outcomes for a measure was expressed as an odds ratio, which is calculated by exponentiating each measure’s logit coefficient. Finally, to assess whether entirely black cats fared differently during the month of Halloween, monthly outcomes for a subsample of 1219 entirely black cats were juxtaposed using a global chi-square test, as well as *z* tests for specific contrasts between months on each outcome. Significance tests in this study used *α* = 0.05. Analyses were conducted using IBM SPSS Statistics, Version 26 and Stata/SE 15.1.

## 3. Results

The most frequent outcome by far was euthanasia (see Table 1): there were 5649 euthanizations (70.8%), 1048 adoptions (13.1%), and 1286 transfers (16.1%). The most and least common colors were black and “other” at 2641 (33.1%) and 194 (2.4%), respectively. Table 1 also provides contingency tables for coat color by cat outcome, as well as for color by each of two controls. Frequencies and column percentages are presented, with statistical equivalence (using *z* tests) for colors depicted with superscripted letters. As shown by a superscripted “a,” black cats had the highest rate of euthanasia (74.6%) and the lowest rate of adoption (10.0%) of any color. Gray and orange had similar rates of euthanasia (71.8% and 71.1%) and adoption (13.6% and 13.9%), respectively. Brown exhibited a euthanasia percentage similar to that of orange (67.6% versus 71.1%), and its adoption rate (13.1%) was similar to those for both orange and gray. White shared the distinction of lowest euthanasia rate (63.0%) with cats of “other” color, as well as the highest adoption rate (18.8%). In terms of the transfer outcome, black, gray, orange, and other had the lowest percentages (14.7% to 17.3%), while brown and white held the highest (19.3% and 18.2%, respectively). Although the global relationship between coat color and cat outcome was statistically significant *Χ*^2^_(10)_ = 79.44 (*p* < 0.001), its Cramer’s V of less than 0.10 (V = 0.07) suggesteda negligible difference [38]. In terms of pairwise comparisons, black versus white yielded a small association (Cramer’s V = 0.12, not shown in tabular format), while all remaining comparisons were negligible (Cramer’s V < 0.10). Before the introduction of breed and transfer status as controls, black cats generally had the worst outcomes (highest euthanasia and lowest adoption percentages); brown, gray, and orange had middling outcomes; and white and “other” colors had the most favorable outcomes (highest adoption and lowest euthanasia percentages).

Table 1 also provides a breakdown of coat color by two controls, breed and transfer status. White and “other” were underrepresented among domestic shorthairs and overrepresented among fancy mixes and fancy single breeds. Black, gray, and orange evinced few differences on breed, but brown was less represented among domestic longhairs and more represented among fancy breeds. Cramer’s V = 0.08 (*p* < 0.001) suggested that color had a negligible association with breed. The regression analysis to follow assesses the degree to which breed may mediate the relationships between black and white with respect to cat outcomes. Although some pairwise comparisons of color by stray status were statistically significant, color exhibited a nonsignificant global relationship with being a stray (*p* = 0.055).

Table 2 displays results from a multinomial logistic regression model for coat color as a predictor of cat outcomes, controlling for breed and stray status. Black and domestic shorthairs serve as the referent color and breed, respectively. The effect of each measure is expressed as an odds ratio (OR), which represents either the ratio of the (a) odds of euthanasia to the odds of adoption (top panel), or the (b) odds of transfer versus those of euthanasia (bottom panel). An OR = 1.0 in the top panel of Table 2 would indicate that the predicted odds of euthanasia and adoption were identical; ORs > 1.0 indicate increased odds of euthanasia than adoption, while ORs < 1.0 indicate decreased odds for euthanasia. The OR of 0.788 (*p* = 0.034) for having a brown rather than black coat decreases the odds of euthanasia instead of adoption by 21.2% after controlling for breed and stray status. The 95% confidence interval for brown is 0.63 to 0.98 and does not bound OR = 1; the asterisk next to the OR signals a statistically significant (*p* < 0.05) color contrast. All color contrasts exhibit ORs < 1.0 for euthanasia versus adoption, suggesting disadvantage to having a black coat versus the remaining five colors. In contrast, white cats had significantly lower ORs compared to black, brown, and gray (not shown in tabular format), which suggest that the introduction of the two controls did not appreciably alter those bivariate relationships highlighted in Table 1. However, white and orange cats no longer differed significantly in the controlled model as they did in Table 1. Compared to domestic shorthairs, all breeds had lower ORs for euthanasia versus adoption, with fancy mixes and single breeds exhibiting the lowest odds (ORs = 0.148 and 0.165, respectively, at *p* < 0.001). Finally, being a stray increased the odds of euthanasia by 106% (*p* < 0.001).

Table 2 also shows ORs for transfer against euthanasia. Only brown and white cats had significantly higher odds of transfer (OR = 1.316, *p* = 0.003 and OR = 1.304, *p* = 0.015, respectively) than black cats. All breeds except domestic longhairs (*p* = 0.127) had significantly higher ORs of transfer than shorthairs, with fancy breeds exhibiting the highest odds of transfer (OR = 3.435, *p* < 0.001 for single breeds and OR = 2.775, *p* < 0.001 for mixes). Being a stray decreased the odds of transfer by nearly 24% (*p* < 0.001). The Nagelkerke Pseudo R^2^ was modest at 0.062, suggesting that the model only weakly predicted cat outcomes using these three predictors.

Table 3 summarizes monthly frequencies/percentages for the 1219 entirely black cats over the two-year span. Daggers next to particular frequencies denote months that differed statistically (*p* < 0.05) from October on an outcome. Black cats’ euthanasia percentages during October could not be differentiated from those for seven other months; only March, April, May, and December differed, but these months exhibited lower euthanasia percentages than did October. Black cats’ adoption percentage during October was similar to those for seven other months; only January, February, March, and December differed (with higher percentages) than October. In addition, only April and May had different (higher) transfer percentages than October. Monthly adoption and transfer outcomes should be interpreted cautiously for entirely black cats, as their cell frequencies were modest. Although a sample containing more black cats may have yielded more statistical power to detect monthly differences, this subsample does not show advantageous (or even novel) October shelter outcomes for black cats relative to other months.

## 4. Discussion

This study found mixed support for the importance of coat color (*H*_1_) via three tiers of progressively better euthanasia/adoption outcomes for cats across three color groupings: black, brown and gray, and white. Orange cats had worse outcomes than white cats in the contingency table analysis, but the difference disappeared once controls were added to the regression model. However, the global impact of color on outcomes, and even the strongest pairwise difference involving black and white, was weak in magnitude. Further, relationships between coat color and outcomes were selective rather than each color being linked to a distinctive cat outcome(s). Findings were somewhat supportive of BCB (*H*_2_) in that black cats had the highest euthanasia and lowest adoption rates of any of the six colors. There was also limited evidence for DCS in that there were progressively better euthanasia/adoption outcomes for darker to lighter coat shades: black, brown and gray, and finally, white cats. DCS may also explain the shift in relative positioning of orange cats depending on the model examined; orange coats may be perceived as somewhat lighter than brown and gray while somewhat darker than white, but not sufficiently lighter or darker to fully constitute a fourth tier on cat outcomes. Overall, this study’s findings on color and shade largely dovetail with those of several other studies [30,31,32,34], although [30] found similar adoption rates for black and brown cats. In contrast, other studies have reported no color differentiation on outcomes [13] or no effect on length of stay for black cats [9].

Contextualizing the findings of this study—or that of any other on coat color—with the extant literature is not as straightforward as one might expect due to the lack of a uniformity in color-coding cats. For instance, some studies rely on a dichotomous coding of black versus all others [9,32], a continuum of lighter to darker coat shades [32], color categories [8], and still others directly incorporate coat patterns such as tabby, point, or tortoiseshell [13,30]. Others attempt to distinguish between solid and primary/secondary colors [32]. Solid colors are sometimes complicated by very specific and potentially ambiguous colors, such as beige, tan, and cream, that may not fall neatly into a broader color category, or gray, that might translate into lighter or darker shades. This shmorgasboard of coding makes study comparisons on color difficult, if not potentially misleading, and hampers the possibility of a meta-analysis across studies to summarize what the overall impact of color might be on cat outcomes.

Due to the inconsistent nature of how color is coded, it is then difficult to provide shelters with a unified body of research that may provide some direction on how to improve outcomes. A uniform intake coding system across shelters would be ideal for researchers to analyze the potential impact of physical characteristics on adoption decisions. It may be burdensome for some shelters to increase their data collection and maintenance efforts given that many operate with limited staff and other resources, and many shelters and agencies may be reluctant to make their data available to researchers unless compelled to do so. However, there is great potential for collaboration between researchers and organizations that would collect and share more standardized data. Not only would analytic quality be bolstered with the enhanced measurement of coat characteristics, shelters would be in prime position to help researchers better understand the context in which the data were collected, e.g., community preferences, changing adoption criteria, changes in staffing, new regulations, rehoming programs, or budgetary issues that may undergird findings and thus their interpretation. By making just a few low or no cost changes regarding data collection under Kentucky’s Humane Shelter Law [36], Kentucky could become a national model in data collection and collaborative analysis that would contribute to major improvements in the shelter care of companion animals [4].

Although the strength of the findings regarding color (even involving black cats) were weak at best, it seems important to remember that the outcome studied was literally life or death; even a small reduction in the likelihood of euthanasia seems important if considered from the perspective of a sheltered animal. Further, it is likely that a large number—if not a majority—of these cats were suitable for adoption by way of health and disposition; in these cases, some argue that euthanasia should be considered killing instead, as the former connotes alleviation of suffering, particularly when there is little or no possibility of improvement [40]. For these reasons, this study hints that black cats should be provided extra consideration with respect to rehoming, particularly if such efforts do not substantially redirect resources from other rehoming programs.

Through their attempts to provide engaging visual images of cats in order to provide the best chance of adoption, shelters and other organizations inevitably draw attention to coat color. As mentioned earlier, various strategies have been applied to help black and darker-coated cats to be photographed in an optimal fashion. Yet rehoming programs also often attempt—in a cat’s “bio”—to emphasize characteristics not immediately visible in a photo, such as particular behaviors or preferences that may set a cat apart and be desirable to a particular adopter. Thus, desirable behavioral factors could be used to “offset” any potential effect of a less preferred coat color. Intriguingly, one recent study hints that when potential adopters viewed photographs of dogs on an online Australian adoption directory, they preferred photographs containing contextual information hinting that dogs were more in need of rescue, e.g., non-professional photographs, being seen with fewer trappings of ownership such as collars and toys, and being shown inside the shelter rather than outdoors [41]. The authors speculated that such photograph characteristics might evoke a “caring response” from would-be adopters (p. 10), although the study did not track whether this “caring response” affected adoption behavior. In practice, researchers seldom rank predictors of adoption in order of importance so that shelters can readily use the information strategically in rehoming. For instance, is a black-coated short-haired kitten that is active in play more or less likely to be adopted than a 12-year old primarily white cat with medium length hair that is relatively inactive? Predictors of cat outcomes—in combination—are often modeled as additive effects, but they might operate in a more complex manner wherein the effect of one predictor depends on the level of another, i.e., conditional effects. A greater understanding of potentially nuanced relationships between coat color and other predictors would permit shelters to better allocate their rehoming resources.

Further, this study found no evidence that black cats experienced more favorable shelter outcomes during October, suggesting that Halloween did not improve their fate. Yet it is still possible that the public’s interest in—and demand for—black cats is greater just before Halloween, just as the interest in rabbits increases around Easter [42]. Halloween has enjoyed increased popularity in the United States over the last decade as evidenced by increased numbers of participants and planned spending, and even spending on pets’ costumes [43]; the cultural salience of Halloween may serve to hike the demand for black cats—and could be, as some shelters have already realized, a positive way to promote black cat adoption rather than one that compromises the welfare of adopted/transferred cats, e.g., through participation in occult rituals, serving as merely living decorations, or satisfying only a seasonal interest in a companion animal. Although empirical evidence of cat adoption and subsequent maltreatment linked to Halloween-related themes or traditions does not appear to be commonplace [29], future research should explore this possibility, since it would have obvious implications for Halloween-related promotions of black cats.

According to Andrea Blair, Communications Director at the Kentucky Humane Society (KHS), attempts to limit black cat adoptions in October to discourage their misuse/abuse have the opposite effect since she claims black cats “take two to three times longer to get adopted” and removing them as potential adoptees imposes a “barrier” that decreases their chances of finding “loving homes” [44]. Further, Robin Vincent, Shelter Operations Director, noted that KHS has a “very thorough adoption process” whereby “each potential adopter meets with an adoptions counselor…[to] get to know each adopter, which helps ensure that every pet is adopted into a home that truly wants them” [44].

Finally, future work should aim for a better understanding of the mechanisms by which adoption preferences related to color may arise, which in turn, should help to counter the prevailing image(s) of black cats. Asking potential adopters to list or even rank characteristics they prefer in a companion animal may not fully illuminate underlying mechanism(s) that result in BCB due to potential mismatches between behavioral intentions and actual behaviors, as well as an individuals’ incomplete accounting of their subconscious motivations. However, any additional information about cat characteristics or behaviors could be potentially useful in addressing the overpopulation of cats in shelters.

All empirical studies have limitations, and the present one is no exception. The multinomial logistic regression model statistically controlled for breed and stray status, but it cannot obviate all possible differences at intake between the six color-based groups of cats. For instance, potential controls like sex, age, and health at intake had large numbers of missing values and thus were not included in the model. Further, the coat color categories used were limited by the information recorded by the shelter, e.g., gray was not designated as light or dark. If these and other variables in the study were measured with more granularity they might have strengthened or weakened the observed relationships of coat color with outcomes. Finally, some cell counts for the entirely black subsample were relatively small given that the 1219 cases were stretched across three outcomes and 12 months (refer to Table 3); higher monthly intakes or a longer study timespan might have resulted in more robust cell counts.

## 5. Conclusions

On one hand, coat color was not a strong predictor of cat outcomes in this study, which could be viewed through a favorable lens by those concerned that cats of one coat color or another might be afforded powerful advantage or disadvantage in shelters. On the other hand, consistent with other research on perceptions of black cats as well as their actual outcomes in shelters, we find weak evidence in support of BCB since black cats fared slightly worse on euthanasia and adoption outcomes. Further, entirely black cats did not enjoy improved outcomes during the month prior to Halloween, the one time of year when their outcomes might be expected to improve. That white cats had the most favorable euthanasia and adoption outcomes while brown and gray cats had middling outcomes between those of white and black cats presents intriguing, graduated evidence for DCS. In sum, this study hints that black cats in public shelters should receive extra consideration for rehoming, particularly if such efforts do not substantially redirect resources from other initiatives.

Finally, cat coat color is notoriously difficult to describe given its wide variety, and shelter staff may encounter constraints to recording nuanced measurements, e.g., time, ease of data entry, and perhaps most important, insufficient incentive or standardized training to parse color descriptors. Thus, color assessment is likely subjective and inconsistent even for the same rater. Measurement error on coat color may account for some of the disparate findings between extant studies on this topic. More collaboration is needed between shelters and applied researchers to ensure collection of high-quality data for robust analyses.

## Figures and Tables

**Table 1:** Contingency Tables for Coat Color by Cat Outcome and for Coat Color by Controls (*n* = 7983).

		Coat Color					
Measure	Full Sample	Black (*n* = 2641)	Brown (*n* = 1225)	Gray (*n* = 1940)	Orange (*n* = 1062)	White (*n* = 809)	Other (*n* = 306)
***Cat Outcome***							
Euthanasia	5649	1970 ^a^ 74.6%	828 ^b,c^ 67.6%	1392 ^d^ 71.8%	755 ^c,d^ 71.1%	510 ^e^ 63.0%	194 ^b,e^ 63.4%
Adoption	1048	265 ^a^ 10.0%	161 ^b^ 13.1%	263 ^b^ 13.6%	148 ^b^ 13.9%	152 ^c^ 18.8%	59 ^c^ 19.3%
Transfer	1286	406 ^a,b^ 15.4%	236 ^c^ 19.3%	285 ^b^ 14.7%	159 ^a,b^ 15.0%	147 ^a,c^ 18.2%	53 ^a,b,c^ 17.3%
				Global *Χ*^2^_(10)_ = 79.44 * Cramer’s V = 0.07			
***Controls***							
Breed							
Domestic Shorthair	6667	2247 ^a,b^ 85.1%	1013 ^b^ 82.7%	1635 ^a,b^ 84.3%	911 ^a^ 85.8%	630 ^c^ 77.9%	231 ^c^ 75.5%
Domestic Mediumhair	600	207 ^a^ 7.8%	75 ^a^ 6.1%	148 ^a^ 7.6%	78 ^a^ 7.3%	65 ^a^ 8.0%	27 ^a^ 8.8%
Domestic Longhair	393	140 ^a^ 5.3%	41 ^b^ 3.3%	103 ^a^ 5.3%	53 ^a^ 5.0%	39 ^a,b^ 4.8%	17 ^a,b^ 5.6%
Fancy—Mix	192	31 ^a^ 1.2%	63 ^b^ 5.1%	29 ^a^ 1.5%	15 ^a^ 1.4%	40 ^b^ 4.9%	14 ^b^ 4.6%
Fancy—Single Breed	131	16 ^a^ 0.6%	33 ^b^ 2.7%	25 ^c^ 1.3%	5 ^a^ 0.5%	35 ^d^ 4.3%	17 ^d^ 5.6%
				Global *Χ*^2^_(20)_ = 213.02 * Cramer’s V = 0.08			
Stray	5875	1946 ^a,b^ 73.7%	885 ^b,c^ 72.2%	1464 ^a^ 75.5%	780 ^a,b,c^ 73.4%	567 ^c^ 70.1%	233 ^a,b^ 76.1%
				Global *Χ*^2^_(5)_ = 10.81 Cramer’s V = 0.04			

* *p* < 0.05 (Global *Χ*^2^ test). Note: Superscripted letters (a through e) signify coat colors that did not differ on a measure (*p* > 0.05 for 2-tailed *z* tests).

**Table 2:** Multinomial Logistic Regression of Cat Outcome on Coat Color and Controls.

	Model	
Measure	Odds Ratio	95% C.I.
***Euthansia* vs. *Adoption***		
**Brown ^a^**	0.788 *	0.63 to 0.98
**Gray ^a^**	0.712 *	0.59 to 0.86
**Orange ^a^**	0.677 *	0.54 to 0.84
**White ^a^**	0.536 *	0.43 to 0.68
**Other ^a^**	0.511 *	0.37 to 0.71
Domestic Mediumhair ^b^	0.621 *	0.49 to 0.79
Domestic Longhair ^b^	0.499 *	0.38 to 0.65
Fancy—Mix ^b^	0.148 *	0.11 to 0.21
Fancy—Single Breed ^b^	0.165 *	0.11 to 0.25
Stray	2.058 *	1.79 to 2.37
Intercept	5.213 *	4.43 to 6.13
***Transfer* vs. *Euthanasia***		
**Brown ^a^**	1.316 *	1.10 to 1.58
**Gray ^a^**	0.991	0.84 to 1.17
**Orange ^a^**	1.028	0.84 to 1.26
**White ^a^**	1.304 *	1.05 to 1.62
**Other ^a^**	1.226	0.89 to 1.70
Domestic Mediumhair ^b^	1.429 *	1.15 to 1.78
Domestic Longhair ^b^	1.246	0.94 to 1.65
Fancy—Mix ^b^	2.775 *	1.88 to 4.09
Fancy—Single Breed ^b^	3.435 *	2.20 to 5.37
Stray	0.770 *	0.67 to 0.88
Intercept	0.235 *	0.20 to 0.27
Nagelkerke Pseudo R^2^	0.062	

* *p* < 0.05 (two-tailed Wald test). ^a^ Reference is Black. ^b^ Reference is Domestic Shorthair.

**Table 3:** Contingency Table for Cat Outcome by Month for Entirely Black Cats (*n* = 1219).

Cat Outcome	Jan	Feb	Mar	Apr	May	Jun	Jul	Aug	Sep	Oct	Nov	Dec
Euthanasia	44	40	52†	51†	80†	160	105	107	100	85	67	49†
	73.3%	78.4%	67.5%	60.0%	66.1%	86.5%	78.4%	84.9%	81.3%	83.3%	80.7%	68.1%
Adoption	10†	7†	11†	9	3	10	12	8	9	4	2	14†
	16.7%	13.7%	14.3%	10.6%	2.5%	5.4%	9.0%	6.3%	7.3%	3.9%	2.4%	19.4%
Transfer	6	4	14	25†	38†	15	17	11	14	13	14	9
	10.0%	7.8%	18.2%	29.4%	31.4%	8.1%	12.7%	8.7%	11.4%	12.7%	16.9%	12.5%

Notes: (1) † symbols denote months that differed significantly from October on an outcome (*p* < 0.05, 2-tailed *z* tests). (2) Global *Χ*^2^_(22)_ = 95.65, (*p* < 0.05); Cramer’s V = 0.20 across all three outcomes.

## Appendix A. Frequencies of Fancy Single Breeds (*n* = 131)

Abyssinian	2
American Curl Longhair	4
Bengal	10
Bombay	3
British Shorthair	2
European Shorthair	1
Himalayan	1
Korat	1
Maine Coon	16
Manx	10
Oriental Shorthair	1
Persian	4
Ragdoll	2
Russian Blue	21
Somali	2
Siamese/Balinese	36
Snowshoe	10
Tonkinese	1
Turkish Angora	4
